# Epidemiological Characteristics and Economic Burden of Dengue in Zhejiang Province, China

**DOI:** 10.3390/v15081731

**Published:** 2023-08-13

**Authors:** Yi Yu, Ying Liu, Feng Ling, Jimin Sun, Jianmin Jiang

**Affiliations:** 1 Department of Epidemiology and Biostatistics, School of Public Health, Hangzhou Normal University, Hangzhou 311121, China; yiyu20212021@163.com; 2 Key Lab of Vaccine, Prevention and Control of Infectious Disease of Zhejiang Province, Zhejiang Provincial Center for Disease Control and Prevention, Hangzhou 310051, China

**Keywords:** dengue, economic burden, epidemiological characteristics

## Abstract

Dengue imposes a heavy economic burden on families and society. We used surveillance data reported in 2019 to characterize the dengue epidemic in Zhejiang Province, China, which provided guidance for dengue prevention and control. Dengue epidemics mostly occurred in July to October. People aged 30–44 years, males, and commercial service workers were more likely to suffer from dengue. The epidemic areas were mainly in Hangzhou and Wenzhou. Meanwhile, we assessed the economic cost of dengue in the province from both family and organizational perspectives. The direct economic burden of dengue patients was estimated to be USD 405,038.25, and the indirect economic burden was USD 140,364.90, for a total economic burden of USD 543,213.00. The direct economic burden of dengue patients should be reduced by increasing the coverage and reimbursement of health insurance. Additionally, the total annual cost of dengue prevention and control for the government and organizational sectors was estimated to be USD 7075,654.83. Quantifying the dengue burden is critical for developing disease control strategies, allocating public health resources, and setting health policy priorities.

## 1. Introduction

Dengue is one of the most-widespread arthropod-borne viral diseases in the world and is mainly prevalent in tropical and subtropical regions of the globe with a gradual spread to temperate regions [1,2]. The disease imposes a huge health, economic, and political burden on families and societies. In the past few decades, the frequency, magnitude, and extent of dengue have gradually increased in response to changing ecological, economic, and social factors [3]. It is estimated that more than 2.5 billion at-risk people live in countries and regions where dengue virus is transmitted, such as Southeast Asia, the Pacific Islands, and the Caribbean, and transmission occurs within more than 100 countries [4,5]. The first case of dengue was reported in 1978 in Foshan City, Guangdong Province, China. Since then, dengue cases have been reported in several regions, including Guangdong, Hainan, Yunnan, Fujian, and Zhejiang [6,7]. In recent years, due to climate warming and frequent foreign trade, the spatial distribution of dengue in China has gradually expanded and the number of cases has increased dramatically in many provinces [8]. The year 2019 saw the largest-scale dengue epidemic in China in nearly 20 years, with >22,000 cases reported and involving 13 provinces [9].

Assessing the economic burden of dengue is essential for informing decision-makers, establishing health policy priorities, and implementing disease interventions. The economic burden of dengue can be quantified by the costs to society, both direct and indirect, for the diagnosis, treatment, and prevention of the disease. There are four main areas where the costs are spent: disease diagnosis and treatment, surveillance, control and prevention measures, and management of the outbreak [10]. This implies that stakeholders such as individuals, households, communities, and the government make contributions to the accumulation of dengue-related costs. The total annual cost of dengue worldwide is estimated to be USD 8.9 billion (95% UI: 3.7 billion–19.7 billion) [11]. In Thailand, the average annual cost of dengue is approximately USD 180 million, and the average cost of dengue in Singapore is about USD 90 million per year, with 54.6% for disease control and prevention costs [12,13,14]. According to a 2019 study in China, it was estimated that China invested about USD 450 million in the annual cost of dengue, which exceeds the cost of most dengue-endemic countries.

Zhejiang Province is located in the coastal area of China, which belongs to the subtropical monsoon climate and is suitable for the growth and breeding of Aedes albopictus, one of the vectors of dengue virus. Moreover, the mobility of the population in Zhejiang Province is high, and imported dengue cases are often reported, leading to local outbreaks. The Chinese Center for Disease Control and Prevention classifies Zhejiang Province as one of the high-risk provinces for dengue outbreaks in China [15,16]. Therefore, in this retrospective study, we characterize the prevalence of dengue in Zhejiang Province in 2019 and provide the first analysis of the economic burden of dengue from both family and organizational perspectives to understand the healthcare and prevention costs of dengue in Zhejiang Province. We also provide reference suggestions for the allocation of health resources and intervention planning for prevention and control measures for dengue.

## 2. Materials and Methods

### 2.1. Study Sites

This study analyzed the epidemiological characteristics of dengue case data (920 cases) collected in Zhejiang Province in 2019. The case data of dengue were obtained from the disease control and prevention information system of China. A whole-group sampling method was used in the family perspective of this survey. Based on the reported cases of dengue in Zhejiang Province in 2019, the two cities (Hangzhou and Wenzhou) with the largest number of reported cases were selected as prefecture-level cities in Zhejiang Province, and 340 dengue cases were randomly selected from the survey sites for the study. A two-stage stratified random sampling method was used in the social perspective. The first-stage sampling sites were selected based on the distribution of dengue case types in Zhejiang Province in 2019, and the counties (districts) of the selected cities were divided into two levels for the survey: counties (districts) with imported cases and counties (districts) with local cases, with a number of representative counties (districts) randomly selected in each stratum for the survey. A total of 17 counties (districts) were selected as survey points. Imported cases were those who had visited a dengue-endemic country or region within 14 days before the disease onset. Local cases refer to dengue cases that did not leave the county (district) within 14 days before the onset of the disease. In the second stage, 1-2 streets, governments, and the Centers for Disease Control and Prevention (CDCs) with dengue cases were randomly selected from each of the counties (districts) in the first stage to conduct the survey.

### 2.2. Survey Approach

The economic burden from the family perspective refers to the financial loss borne by patients and their parents/guardians, including direct medical costs (diagnosis, treatment, self-purchased medicines, hospitalization, etc.), direct non-medical costs (transportation, accommodation for care givers, meals and nutrition), and indirect costs (loss of income due to the absence of patients and family members from work because of illness). Direct medical costs were assessed retrospectively by reviewing hospital medical records and medical costs associated with patients treated and diagnosed with dengue collected by the health insurance office. Direct non-medical costs and indirect economic burden were assessed from data collected through questionnaires with dengue patients or their parents/guardians. This study was reviewed and approved by the Ethics Committee of the Zhejiang Provincial Center for Disease Control and Prevention (No. 2020-021). All participants signed a consent form.

Organizational aspects of the economic burden of dengue were investigated, including treatment costs and disease prevention costs for dengue patients by government and other organizations (such as the CDC, local communities, etc.). Dengue prevention and control costs of organizational units in selected counties (districts) were collected through an electronic questionnaire.

### 2.3. Data Analysis

The epidemiological characteristics of dengue cases were analyzed descriptively. For the family perspective on the economic burden of dengue, we calculated the direct economic burden, which is the sum of direct medical, direct non-medical, and indirect medical costs. The indirect economic burden was measured using the human capital approach by collecting the number of days of lost work and disposable income per capita (in days) for patients and their family members. Data on disposable income per capita were obtained from the 2019 Zhejiang Province Statistical Yearbook. Finally, the number of dengue cases in Zhejiang Province in 2019 was multiplied by the economic burden per capita to calculate the family economic burden of dengue. We used the Mann–Whitney U-test (two independent samples) and Kruskal–Walls H-test (three or more independent samples) to analyze the influencing factors of family economic burden. Finally, after a normality test for the variables, a multiple linear regression model was established to explore the association between economic burden and demographic characteristics.

From the perspective of organizational economic burden, we multiplied the median cost of different types of counties (districts) by the number of dengue counties (districts) in the respective categories and added them to the cost of the government’s health insurance scheme to value the total cost spent on dengue prevention and treatment in Zhejiang Province. SPSS 23.0 was used for statistical analysis, and *p* < 0.05 was statistically significant.

## 3. Results

### 3.1. Descriptive Statistics

Dengue cases numbering 920 were reported during 2019 in Zhejiang Province. There were 609 imported cases and 311 local cases. The demographic characteristics of the patients are summarized in Table 1. Among all dengue cases, there were 562 males and 358 females. Males accounted for the majority of patients (562; 61.09%). However, a much smaller proportion of imported cases were female than local cases (33.50% vs. 49.50%). Age ranged from 8 months to 87 years (median 41 years). The largest number of patients were in the 30–44 years age group (343; 37.28%), followed by 45–59 years (273; 29.67%) and 15–29 years (168; 18.26%). Among them, the age of imported cases was mainly concentrated in the age group of 15–59 years (562; 92.29%), while the age distribution of local cases was between 30 and 69 years (266; 72.66%). As shown in Table 1, dengue cases in 2019 occurred mainly in the commercial service provider group, and the proportion involved in commercial service providers was 20.54%. This was followed by farmers and domestic, housework, and non-working. Notably, the local cases occurred mainly in the domestic, housework, and non-working and worker groups, with 21.54% and 16.40% of local cases, respectively.

**Figure 1:**
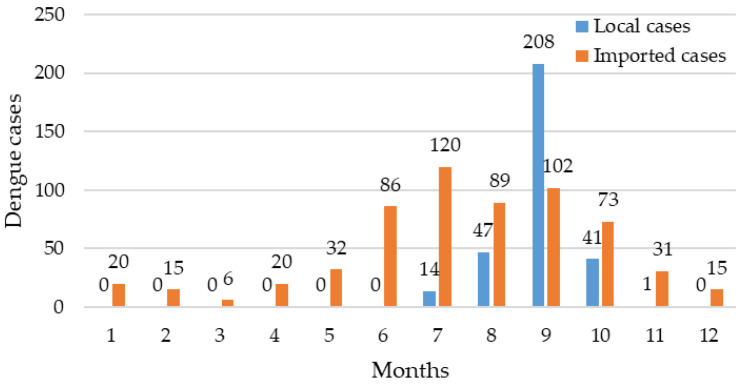
Monthly distribution of dengue cases in Zhejiang Province in 2019.

The monthly distribution of dengue cases in Zhejiang Province in 2019 is shown in Figure 1. A total of 920 dengue cases were recorded, and the average monthly number of dengue cases was 77, ranging from 6 cases (March) to 310 cases (September). As shown in Figure 1, the epidemiological situation of imported dengue cases was relatively stable, with a plateau period from June to October. The number of imported dengue cases in July was the highest in 2019 in Zhejiang Province, with 120 cases. Imported cases of dengue were reported in Zhejiang Province every month. The monthly distribution of dengue revealed that most local cases were reported between July and October, with a marked increase in the number of cases in September.

**Figure 2:**
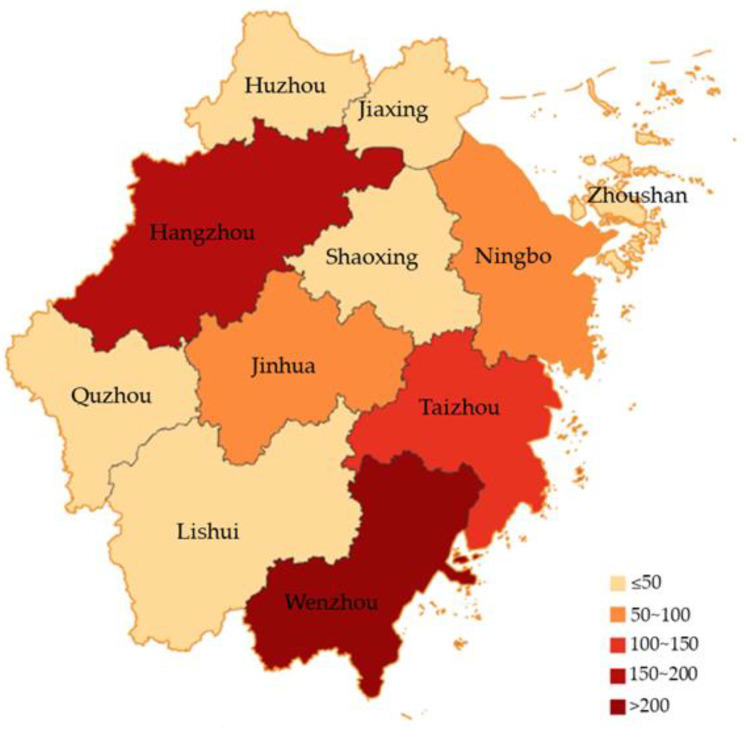
Regional distribution of dengue reports in Zhejiang Province in 2019.

**Figure 3:**
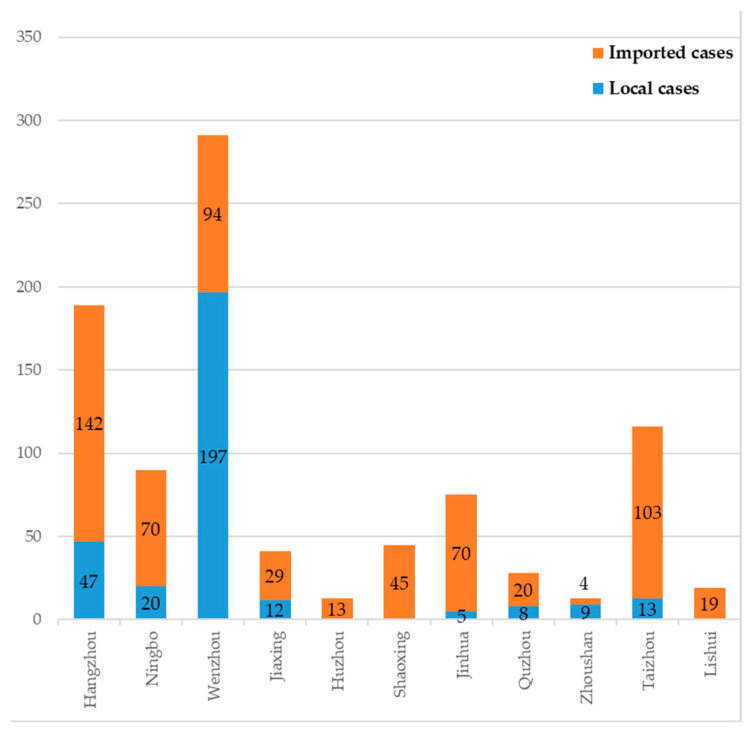
Geographical distribution of dengue cases in Zhejiang Province by imported and local cases.

The geographic distribution of dengue cases in Zhejiang Province in 2019 is represented in Figure 2. We summarized the dengue cases of 11 prefecture-level cities in Zhejiang Province, with the top two cities with the highest cumulative number of cases being Wenzhou (291; 31.63%) and Hangzhou (189; 20.54%), accounting for 52.17% of the total number of dengue cases (Figure 2). As shown in Figure 3, we also observed that imported cases were reported in all prefecture-level cities during 2019. Hangzhou reported the highest number of imported cases at 142 (23.32%), followed by Taizhou (103; 16.91%). The number of cities affected by local cases was fewer than imported cities: eight cities were affected, with Wenzhou being the most affected (197; 63.34%).

### 3.2. Family Economic Burden of Dengue Patients

A total of 340 patient records were reviewed for the retrospective, direct medical cost calculation (126 in Hangzhou and 214 in Wenzhou). The distribution of patients with different types of dengue is shown in Table 2. Among the 340 patients, 39 were outpatients (11.47%) and 301 were inpatients (88.53%). There were 197 males (57.94%) and 143 females (42.06%), with a medium age of 39 years. There were 299 patients who had no basic illness prior to dengue (87.94%). There were 278 patients that were covered by any type of medical insurance (81.76%).

### 3.3. Family Perspective Economic Costs

**Figure 4:**
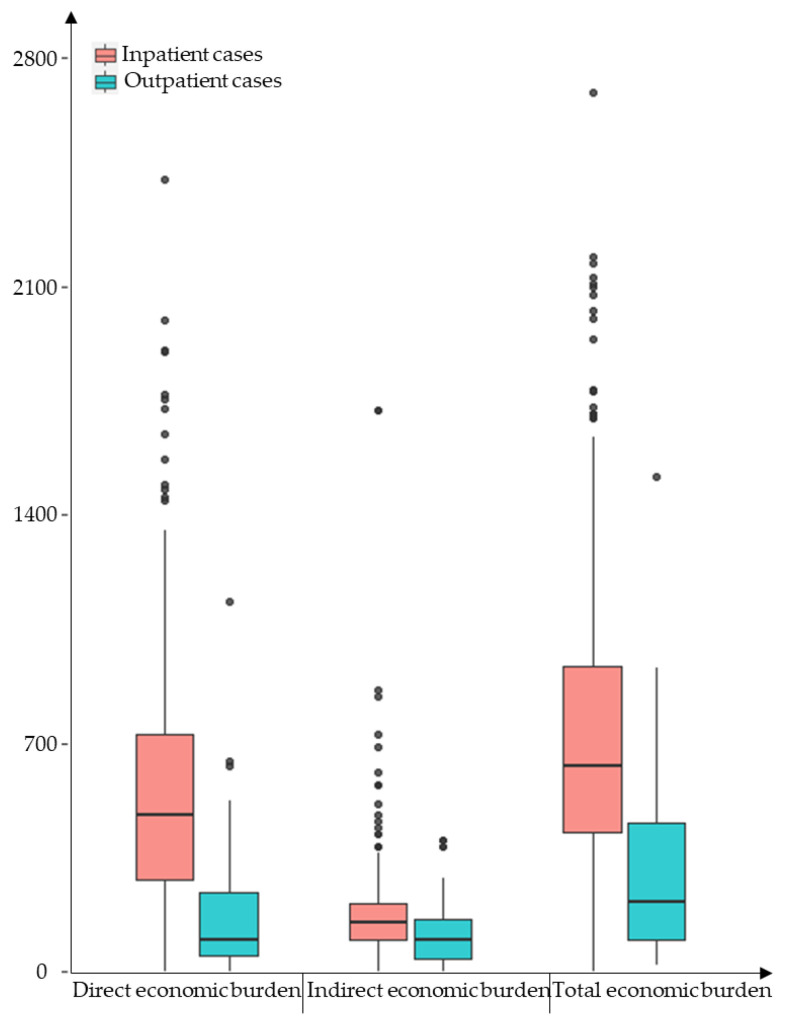
Economic burden of disease per capita for dengue cases in Zhejiang Province, 2019 (USD).

Among the 340 dengue patients, the median direct economic burden, indirect economic burden, and total economic burden were USD 440.26, USD 152.57, and USD 590.45 respectively (Figure 4). The median direct economic burden for outpatients and inpatients was USD 99.32 and USD 478.35, respectively; the indirect economic burden was USD 95.36 and USD 152.57, respectively; the total economic burden was USD 213.57 and USD 629.56 (Table 3). In the province, the direct economic burden of dengue in 2019 was estimated to be USD 405,038.25$ and the indirect economic burden was USD 140,364.90, for a total economic burden of USD 543,213.00.

In this study, we analyzed the factors influencing the economic burden of dengue patients (Table 4). The Mann–Whitney U-test and Kruskal–Walls H-test found that type of case diagnosis, household income, place of residence, type of health insurance, and number of lost days from work significantly influenced the total economic burden of dengue patients (*p* < 0.05). In addition, the difference in the economic burden of indirect illness for dengue patients was also statistically significant for age (*p* = 0.028), and the effect of basic illness on indirect economic burden was slightly non-significant (*p* = 0.091).

To further investigate the variables affecting economic burden, a multiple linear regression model was conducted. Variables in the univariate analysis were included as independent variables in the multivariate regression model for analysis. The detailed outcomes of the regression model are listed in Table 5. Overall, direct economic burden was associated with age, place of residence, basic illness, type of case diagnosis, lost working days, and medical insurance reimbursement rate (*p* < 0.05). The lost working days variable had a statistically significant association with the indirect economic burden (*p* < 0.05). The results also showed that the factors of total economic burden were place of residence, household income, basic illness, type of case diagnosis, lost working days, and medical insurance reimbursement rate (*p* < 0.05).

### 3.4. Comparing the Dengue Economic Burden from the Organizational Perspective and the Family Perspective

The study found that health insurance costs (new rural cooperative medical, basic medical insurance for urban residents, urban employee insurance, commercial insurance, and others) accounted for a large proportion of the investment in dengue expenditures. From the family perspective, the economic burden of dengue patients gradually decreased as the proportion of health insurance reimbursement increased, with a statistically significant difference (Table 6). From the organizational perspective, it was estimated that the economic burden of dengue cases was mainly due to the costs reimbursed by national health insurance. The average economic burden of patients in cases was USD 200.46. The total cost of treatment for dengue patients assumed by Zhejiang Province in 2019 was estimated to be USD 184,140.

### 3.5. Investment in the Prevention and Treatment of Dengue

In this study, the counties (districts) with imported cases included 6 counties (districts) in Zhejiang Province and with local cases included 11 counties (districts) in Zhejiang Province. Expenses for the prevention and control of dengue at all levels and departments are shown in Table 7. The total economic burden of dengue on counties (districts) with imported cases and counties (districts) with local cases were USD 23,436.00 and USD 123,114.33, respectively.

In Zhejiang Province, 34 counties (districts) had imported cases and 51 counties (districts) had local cases distributed in 2019. In addition to the costs reimbursed by the health insurance, the annual total cost of dengue for prevention and control in Zhejiang Province was estimated at USD 7,075,654.83.

## 4. Discussion

### 4.1. Epidemic Characteristics of Dengue

In our study, the number of imported dengue cases was high, which may be closely associated with the increased intensity and extent of dengue epidemics overseas. According to the World Health Organization, it was found that 2019 was the highest number of reported dengue cases worldwide, and all regions were affected [17]. As a relatively economically developed coastal region, Zhejiang Province has a large population movement, a more developed tourism industry, and frequent transportation traffic; these increase the possibility of imported dengue cases from abroad, as well as the economic cost of preventing, controlling, and treating dengue [18]. Imported dengue cases are an important influencing factor affecting the outbreak of dengue in China [19]. Health education on dengue should be made available to people entering and leaving the country, and surveillance and tracking of infectious diseases should be strengthened to minimize the disease burden caused by dengue.

In terms of gender, males are more susceptible to dengue virus, which may be due to (1) differences in the social division of labor, leading to the greater exposure of males to dengue. The Chinese workforce is heavily male-dominated in industries such as transportation and construction [20,21]. (2) Differences in disease severity between men and women influence treatment-seeking behavior, and such behavioral differences may be responsible for the higher number of reported cases among men [22]. In addition, people aged 30–44 years are more likely to be infected with the dengue virus, which may be due to the working population of young and middle-aged people, who are more prone to increased risk of exposure to infectious diseases due to travel or trade exchanges to dengue endemic areas, which is consistent with previous findings [6,23]. For occupation, the reported cases of dengue in Zhejiang Province in 2019 mainly occurred in the commercial service worker population, which is similar to the findings in Guangdong Province, China [24], suggesting that, due to economic globalization, commercial service workers have more opportunities for overseas trade exchange and cooperation, and people are also at higher risk of dengue infection.

From this study, we observed that local dengue cases in Zhejiang Province appeared from July to October, with a significant seasonality. Imported cases were reported monthly and mainly concentrated in June to October. This is highly compatible with the months of dengue outbreak in Mainland China [25,26,27]. There are studies suggesting that the climatic conditions such as temperature and humidity from July to October are favorable for mosquito breeding, which leads to an increase in the number and density of mosquito vectors and an enhanced intensity of dengue transmission [26,28]. Therefore, we should strengthen mosquito vector surveillance efforts and take preventive and control measures for infectious diseases during the dengue epidemic months.

### 4.2. Dengue Imposes a Huge Economic Burden on Families in the Study Area

The economic burden of dengue may be associated with the regional economic situation. The total per capita cost of dengue cases in this study was CNY 4232.61 in 2019, which was 3.93% of the per capita Gross Domestic Product (GDP) in Zhejiang Province during the same year. A study in Guangdong Province showed that the average cost of dengue cases was about 3.71% of the per capita GDP in the region [29]. The level of healthcare services and medical costs will be higher in areas with relatively developed economies, and the cost of disease treatment will account for a larger proportion of the total dengue cost [30]. In addition, residents in areas with high levels of economic development have higher incomes, have a greater awareness of healthcare for the disease and are more willing to bear the cost of disease prevention [31].

In this study, it was found that the direct cost of dengue in Zhejiang Province played a greater role than the indirect cost, with the direct economic burden representing about 74.56% of the total cost. Meanwhile, another study found that the direct costs of dengue accounted for 60.61% of the total costs in Ningbo [32]. However, a study in Taiwan showed that the indirect costs of dengue were higher than the direct costs [33]. The possible explanation is that the indirect economic burden of dengue in Taiwan is mainly caused by the lack of productivity from fatal cases, whereas no fatal cases occurred in Zhejiang Province in 2019; therefore, the economic burden of the disease mainly derives from the diagnosis and private treatment of the illness. The results of a study on dengue costs in Thailand differed from our study in that indirect costs were the greatest burden in Thailand, which may be due to variations in the healthcare systems of different countries [34]. Thailand has implemented a policy of full coverage of the population-wide healthcare system, which greatly reduces the costs of diagnosis and treatment for dengue patients and mitigates the direct economic costs of dengue. In addition, our study found that the economic burden of the disease, especially the direct cost, decreases with higher reimbursement rates from health insurance, which is consistent with a previous study [32]. It is recommended that the participation rate and reimbursement rate of health insurance should be increased to reduce the household economic burden of dengue.

The study also found that the dengue economic burden borne by the different case types differed significantly. The average costs for outpatient and inpatient cases were USD 217 and USD 639, respectively, with a ratio of 1:4.8, suggesting that the total cost of dengue for families with inpatients is much higher than for outpatients. This may be due to (1) inpatients having more severe symptoms, greater demand for medical resources, and higher costs based on disease detection, treatment, and nursing rehabilitation than outpatients and (2) inpatients spending more time losing work than outpatients, so the indirect costs due to lost productivity will be higher. Therefore, early diagnosis, rapid treatment, and active health education would help reduce the cost of hospitalized cases and also reduce the household economic burden of dengue. In 2010, the per capita cost of dengue hospitalization in Brazil was USD 516 [35]. A study of the economic burden of dengue outpatient and inpatient cases in 2017 found that the total per capita cost of inpatient cases in three dengue-endemic countries, Vietnam, Thailand, and Colombia, had a maximum of USD 385 and a minimum of USD 141, with outpatient costs ranging from USD 40 to USD 158 [34]. They were all lower than the median cost in this study (outpatient: USD 217; inpatient: USD 639), suggesting a higher economic burden of dengue in Zhejiang Province.

In addition to the type of case diagnosis and Medicare reimbursement rates, factors such as age, place of residence, having or not a basic illness, and lost workdays also had an impact on the economic burden of dengue. Our findings showed that the direct economic burden decreased with age, which is different from previous studies [29,36]. This may be due to less exposure of the elderly in dengue-endemic areas, reduced exposure to the virus and mosquito vectors. We also revealed that the direct and total costs of dengue were higher for dengue patients living in rural areas and with underlying diseases. This is probably due to (1) most people living in rural areas selecting rural health centers for medical care (one study found that the level of medical costs in township hospitals is on average higher than in city-level hospitals [37,38]) and (2) dengue patients with basic illness being in poorer physical condition and more likely to have complications, which increase the cost of treatment.

### 4.3. Governments and Organizations Rationally Allocate Dengue Prevention and Control Costs

From the organizational perspective, the dengue economic burden was approximately USD 7075,654.83 in Zhejiang Province in 2019, accounting for about 1.59% of the economic burden of dengue in China in the same year [31]. The cost of dengue in counties (districts) with imported cases is mainly incurred by streets and communities, and the economic burden of dengue in counties (districts) with local cases is mainly borne at the government department level. This indicates that the regular surveillance of imported infectious disease cases from abroad is more important at the street and community level, and that case monitoring should be more intensive, which will consume more human, material, and monetary costs. At the same time, community and street health service centers cost more for mosquito vector control and other measures. The government departments are more burdened with the prevention of infectious diseases such as dengue knowledge dissemination and mosquito breeding environment management. Our study provides some reference for the working focus and funding allocation of dengue prevention and control in Zhejiang Province. For counties and districts with mainly imported cases, community and street surveillance of imported cases and the proportion of funding for mosquito vector control should be increased. Likewise, for counties and districts with local cases, funding should be focused on and strengthened for dengue health education and mosquito vector breeding environment management. Quantifying the burden of dengue is essential for developing disease control strategies, allocating public health resources, and setting health policy priorities.

Recall bias may occur in questionnaire surveys when collecting case information from patients and their families. Secondly, when investigating the economic burden from the organizational perspective, the data content of individual districts (counties) was not complete. Finally, there are differences in the content and items covered by the organizational sector for dengue costs in each province, making it difficult to directly compare the results from the organizational perspective with other studies.

## 5. Conclusions

In this study, we analyzed the epidemiological characteristics of dengue in Zhejiang Province in 2019. The dengue epidemic occurred mostly in July-October. People aged 30-44 years, males, and commercial service workers were more susceptible to dengue. The epidemic areas were Hangzhou and Wenzhou. These findings will help to develop measures and policies for the prevention and control of dengue. Dengue imposes a significant economic burden on individuals, families, and society. This study investigated the economic costs of dengue in the major endemic areas of Zhejiang Province, with the aim of analyzing in depth of the medical and management costs of dengue from a household and organizational perspective. For effective prevention and management of infectious diseases, firstly, border screening should be strengthened and improved, and multisectoral and regional cooperation mechanisms should be promoted for timely detection and follow-up surveillance. Second, increasing the scope of the population and the percentage of the reimbursement amount of health insurance can help reduce the burden on families. Third, health education should be targeted at susceptible populations to ensure early prevention. Fourth, investment in primary health service costs should be strengthened, and training of medical professionals should also be enhanced to improve the ability to identify, diagnose, and treat dengue, to ensure early diagnosis and timely treatment. Finally, priority management should be targeted at areas with a high number of cases to prevent localized transmission and outbreaks of dengue.

## Figures and Tables

**Table 1:** Demographic characteristics of reported dengue cases in Zhejiang Province, 2019.

Characteristics	Local Cases		Import Cases		Total Cases	
	Number of Cases	Percentage (%)	Number of Cases	Percentage (%)	Number of Cases	Percentage (%)
Gender						
Male	157	50.50%	405	66.50%	562	61.09%
Female	154	49.50%	204	33.50%	358	38.91%
Age groups (years)						
0~14	8	2.57%	11	1.81%	19	2.07%
15~29	45	14.47%	123	20.20%	168	18.26%
30~44	79	25.40%	264	43.35%	343	37.28%
45~59	98	31.51%	175	28.74%	273	29.67%
60~69	49	15.76%	34	5.58%	83	9.02%
70~79	26	8.36%	2	0.33%	28	3.04%
≥80	6	1.93%	0	0.00%	6	0.65%
Profession						
Students	6	1.93%	24	3.94%	30	3.26%
Workers	51	16.40%	66	10.84%	117	12.72%
Farmers	37	11.90%	130	21.35%	167	18.15%
Cadres and employees	15	4.82%	28	4.60%	43	4.67%
Retired staff	23	7.40%	18	2.96%	41	4.46%
Domestic, housework, and non-working	67	21.54%	69	11.33%	136	14.78%
Commercial service providers	37	11.90%	152	24.96%	189	20.54%
Others	75	24.12%	122	20.03%	197	21.41%

**Table 2:** General information of dengue cases.

Item	Classification	Count	%
Gender	Male	197	57.94%
	Female	143	42.06%
Age	0~	6	1.76%
	10~	10	2.94%
	20~	41	12.06%
	30~	90	26.47%
	40~	77	22.65%
	50~	63	18.53%
	60~	40	11.76%
	≥70	13	3.82%
Place of residence	Urban	272	80.00%
	Rural	68	20.00%
Household income	<15,000	20	5.88%
	15,000–50,000	72	21.18%
	50,000–100,000	110	32.35%
	100,000–150,000	69	20.29%
	>150,000	69	20.29%
Basic illness	Have	41	12.06%
	None	299	87.94%
Type of medical insurance	Public medical	10	2.94%
	New rural cooperative medical	130	38.24%
	Basic medical insurance for urban residents	41	12.06%
	Urban employee insurance	86	25.29%
	Commercial insurance	5	1.47%
	Other	6	1.76%
	None	62	18.24%
Type of case diagnosis	General outpatient cases	39	11.47%
	General inpatient cases	299	87.94%
	Critically ill hospitalized patients	2	0.59%

**Table 3:** Economic burden of disease per capita for dengue cases in Zhejiang Province, 2019 (USD).

Item	Outpatient Cases M	Inpatient Cases M	Total M
Direct medical costs	78.82	338.15	308.23
Direct non-medical costs	4.19	111.60	99.05
Direct economic burden	99.32	478.35	440.26
Indirect economic burden	95.36	152.57	152.57
Total economic burden	213.57	629.56	590.45

**Table 4:** Economic burden among different types of dengue patients (USD).

Item	Classification	Direct Economic Burden			Indirect Economic Burden			Total Economic Burden		
		Median	Statistic	*p*-Value	Median	Statistic	*p*-Value	Median	Statistic	*p*-Value
Type of case diagnosis	Outpatient cases	99.32	6.98	<0.001	95.36	3.011	0.003	213.57	6.618	<0.001
	Inpatient cases	478.35			152.57			629.56		
Gender	Male	448.00	0.333	0.739	153.12	0.653	0.514	598.79	0.522	0.602
	Female	433.16			133.98			589.11		
Age	0~	475.23	7.984	0.334	143.54	15.737	0.028	708.79	11.127	0.133
	10~	703.99			153.12			935.17		
	20~	497.84			153.12			659.40		
	30~	432.18			133.98			556.39		
	40~	427.00			153.12			633.55		
	50~	461.58			153.12			575.95		
	60~	333.83			95.70			439.78		
	≥70	601.58			133.98			601.58		
Place of residence	Urban	399.42	−4.153	<0.001	133.98	−1.431	0.152	552.89	−3.875	<0.001
	Rural	576.24			153.12			743.85		
Household income	<15,000	607.18	17.324	0.002	95.70	15.362	0.004	736.29	13.2	0.01
	15,000–50,000	497.42			162.68			703.86		
	50,000–100,000	445.48			153.12			602.83		
	100,000–150,000	459.48			133.98			582.90		
	>150,000	326.06			153.12			474.05		
Basic illness	Have	423.22	0.878	0.38	133.98	−1.691	0.091	536.48	−0.026	0.979
	None	442.26			153.12			598.79		
Type of health insurance	Public medical	376.95	71.59	<0.001	133.98	7.977	0.24	424.80	63.186	<0.001
	New rural cooperative medical	548.73			153.12			736.29		
	Basic medical insurance for urban residents	284.90			133.98			476.29		
	Urban employee insurance	247.59			133.98			390.17		
	Commercial insurance	480.06			191.39			676.86		
	Other	305.62			143.54			505.23		
	None	601.44			153.12			758.36		
Lost days	0~	291.06	42.023	<0.001	0.00	324.881	<0.001	306.46	113.452	<0.001
	3~	300.44			95.70			376.65		
	6~	412.23			133.98			551.32		
	9~	512.40			191.39			711.07		
	≥12	689.78			287.09			1102.67		

**Table 5:** Regression of economic burden for dengue sample.

Item	Direct Economic Burden		Indirect Economic Burden		Total Economic Burden	
	β	*p*-Value	β	*p*-Value	β	*p*-Value
Gender	0.03	0.508	−0.022	0.587	0.017	0.672
Age	−0.104	0.045	0.033	0.474	−0.076	0.102
Place of residence	0.127	0.005	0.08	0.05	0.137	0.001
Household income	−0.072	0.144	−0.079	0.073	−0.09	0.041
Basic illness	−0.191	<0.001	0.053	0.211	−0.142	0.001
Type of case diagnosis	0.218	<0.001	−0.533	0.594	0.177	<0.001
Type of medical insurance	−0.046	0.323	0.037	0.38	−0.025	0.543
Lost days	0.27	<0.001	0.678	<0.001	0.479	<0.001
Medical insurance reimbursement rate	−0.35	<0.001	−0.029	0.492	−0.307	<0.001
R2	0.345		0.47		0.473	
F	20.819 ***		34.362 ***		34.716 ***	

*** *p* < 0.001.

**Table 6:** k-w test for different health insurance reimbursement rates and economic burden of illness (USD).

Reimbursement Ratio	Direct Medical Costs	Direct Non-Medical Costs	Direct Economic Burden	Indirect Economic Burden	Total Economic Burden
0~	434.07	102.9	580.16	153.1152	776.2748
20%~	413.28	102.9	529.9	153.1152	665.1358
40%~	340.27	135.94	486.43	133.9758	597.0076
60%~	201.74	72.1	282.73	143.5448	429.3842
80~100%	53.9	84	182	133.9758	317.6558
*p*-value	<0.001	0.071	<0.001	0.14	<0.001

**Table 7:** Organizational cost for prevention and treatment of dengue (USD).

Item	Counties (Districts) with Imported Cases			Counties (Districts) with Local Cases		
	M	Q1	Q3	M	Q1	Q3
Government	15,803.61	1280.13	28,700.00	280,933.47	43.05	725,080.30
CDC	15,853.83	14,115.50	21,832.83	86,086.85	30,569.00	147,497.00
Street office, community	65,230.20	23,738.40	210,381.50	268,519.92	118,554.10	473,146.87
Total	23,436.00	8652.00	48,384.00	123,114.33	29,711.50	292,892.36

## Data Availability

The research data are available upon reasonable request.
